# Molecular Basis for the Neutralization of Tumor Necrosis Factor α by Certolizumab Pegol in the Treatment of Inflammatory Autoimmune Diseases

**DOI:** 10.3390/ijms18010228

**Published:** 2017-01-23

**Authors:** Jee Un Lee, Woori Shin, Ji Young Son, Ki-Young Yoo, Yong-Seok Heo

**Affiliations:** Department of Chemistry, Konkuk University, 120 Neungdong-ro, Gwangjin-gu, Seoul 05029, Korea; jaspersky@naver.com (J.U.L.); woolishin@nate.com (W.S.); jieyson@hanmail.net (J.Y.S.); kiyoung123@daum.net (K.-Y.Y.)

**Keywords:** certolizumab pegol, TNFα, inflammatory bowel diseases, rheumatoid arthritis, therapeutic antibody, crystal structure

## Abstract

Monoclonal antibodies against TNFα, including infliximab, adalimumab, golimumab, and certolizumab pegol, are widely used for the treatment of the inflammatory diseases such as rheumatoid arthritis and inflammatory bowel disease. Recently, the crystal structures of TNFα, in complex with the Fab fragments of infliximab and adalimumab, have revealed the molecular mechanisms of these antibody drugs. Here, we report the crystal structure of TNFα in complex with the Fab fragment of certolizumab pegol to clarify the precise antigen-antibody interactions and the structural basis for the neutralization of TNFα by this therapeutic antibody. The structural analysis and the mutagenesis study revealed that the epitope is limited to a single protomer of the TNFα trimer. Additionally, the DE loop and the GH loop of TNFα play critical roles in the interaction with certolizumab, suggesting that this drug exerts its effects by partially occupying the receptor binding site of TNFα. In addition, a conformational change of the DE loop was induced by certolizumab binding, thereby interrupting the TNFα-receptor interaction. A comprehensive comparison of the interactions of TNFα blockers with TNFα revealed the epitope diversity on the surface of TNFα, providing a better understanding of the molecular mechanism of TNFα blockers. The accumulation of these structural studies can provide a basis for the improvement of therapeutic antibodies against TNFα.

## 1. Introduction

Tumor necrosis factor superfamily (TNFSF) proteins mediate a diverse range of signaling events, including cell growth, survival, and apoptosis, and modulate inflammation, host defense, and organogenesis of the immune, ectodermal, and nervous systems [1,2,3]. The binding of TNFSF proteins to their receptors (TNFRSF) initiates many pro-inflammatory immune responses. It has been known that there are more than 35 specific ligand-receptor pairs between TNFSF and TNFRSF [4]. Among them, TNFα is a major inflammatory cytokine with a crucial role in the pathogenesis of inflammatory autoimmune diseases via interactions with its cognate receptors, TNFR1 and TNFR2 [5,6,7]. TNFα is a trimeric transmembrane protein; it can be cleaved to release a soluble trimer [8,9]. Both a mature form of soluble TNFα as well as a precursor form of transmembrane TNFα can mediate various inflammatory responses [10,11]. Each protomer of a TNFα trimer is formed by a sandwich of an inner and outer β-sheet with all 10 strands [12].

Biological agents against TNFα have been developed for the treatment of inflammatory diseases, including rheumatoid arthritis, psoriatic arthritis, axial spondyloarthritis, and inflammatory bowel diseases, such as Crohn’s disease and ulcerative colitis [13,14,15,16]. The USA Food and Drug Administration (FDA) has approved five TNFα blockers. Four are antibody-based drugs, i.e. infliximab, adalimumab, certolizumab-pegol, and golimumab, and the other drug, etanercept, is a fusion protein composed of two extracellular domains of TNFR2 and the Fc region of IgG1 [17,18,19,20,21]. All of these TNFα blockers bind to both a soluble and a transmembrane form of TNFα, thereby interrupting the TNFα–TNFR interaction [22,23].

Certolizumab pegol has a unique structure compared to those of the other approved therapeutic antibodies against TNFα. It is a monovalent Fab fragment of a humanized anti-TNFα antibody and lacks the Fc region [24]. The hinge region of certolizumab is attached to two cross-linked chains of a 20-kDa polyethylene glycol (PEG) and is therefore named the certolizumab pegol [25]. The lack of the IgG Fc region can result in the fast degradation of biologics because the binding of the Fc region to the neonatal Fc receptor (FcRn) in the endosome is important for regulating antibody homeostasis by protecting IgG from degradation, thereby contributing to the long plasma half-life of IgG [26,27]. However, the plasma half-life of certolizumab pegol is prolonged by the presence of the covalently linked PEG moiety, as PEGylation increases the plasma half-life and solubility and reduces immunogenicity and protease sensitivity [28]. Indeed, the serum half-life of certolizumab pegol (14 days) is comparable to those of other IgG1 drugs, including infliximab (8–10 days), adalimumab (10–20 days), and golimumab (9–15 days), despite its inability to bind to FcRn [29,30]. The distribution of certolizumab pegol in the inflamed joint is greater than those of infliximab and adalimumab [31], and this is probably due to the unique structure of certolizumab pegol. The lack of the Fc region in certolizumab pegol results in no activity of complement-dependent cytotoxicity (CDC) and antibody-dependent cell-mediated cytotoxicity (ADCC), whereas other TNFα blockers can induce potent CDC and ADCC [32,33].

The crystal structures of TNFα-TNFR2 and TNFβ-TNFR1 complexes have established the foundations of the ligand-receptor interactions between TNFSF and TNFRSF, providing invaluable information for understanding the molecular mechanisms of TNF signaling [34,35]. Recently, the crystal structures of TNFα in complex with the Fab fragments of infliximab and adalimumab have been reported, clarifying their epitopes and inhibitory mechanisms by overlap with the TNFα–TNFR interface [36,37]. In the TNFα-infliximab structure, the Fab fragment interacts with only one TNFα protomer in the TNFα trimer, and the EF loop plays a pivotal role in infliximab recognition by TNFα. In the TNFα-adalimumab complex structure, the epitope consists of two adjacent TNFα molecules in the homotrimer of TNFα and is highly similar to the interface of the TNFα–TNFR2 complex. To elucidate the molecular mechanism and epitope of another anti-TNFα agent, certolizumab pegol, we determined the crystal structure of TNFα in complex with the Fab fragments of certolizumab pegol. We examined the binding mode of the complex and the conformational changes induced by antibody binding, thereby clarifying the molecular basis by which certolizumab pegol effectively blocks TNFα-TNFR interactions, despite the monovalency originating from its unique structure.

## 2. Results

### 2.1. Crystal Structure of TNFα in Complex with Certolizumab Fab Fragment

**Figure 1:**
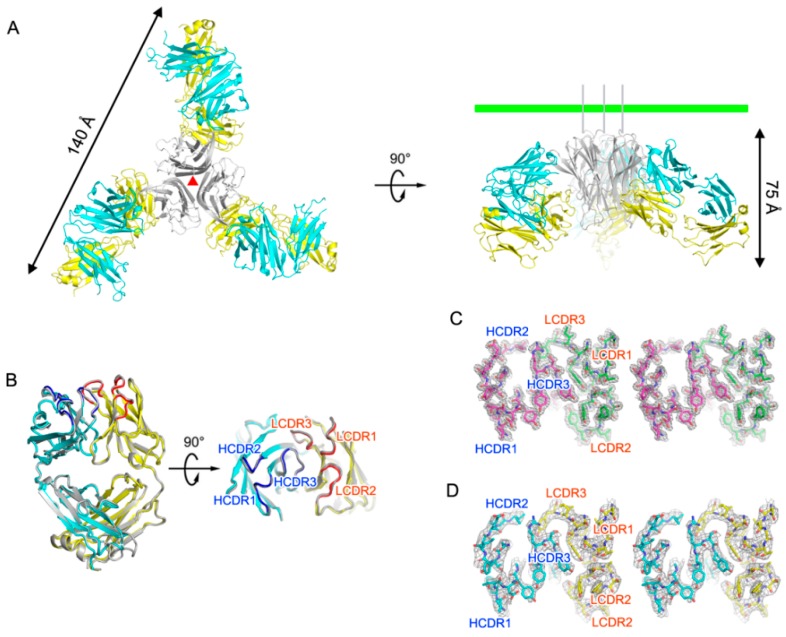
Overall structure of TNFα in complex with the certolizumab Fab fragment. (**A**) Ribbon representation of TNFα (gray) in complex with the certolizumab Fab fragment (heavy chain: cyan; light chain: yellow) in two orientations. The 3-fold axis in the trimeric complex is indicated as a red triangle. The green bar indicates a putative membrane of a TNFα-producing cell if the TNFα trimer is a precursor form of transmembrane TNFα; (**B**) Superposition of the free certolizumab Fab fragment (gray) onto the Fab fragment extracted from the TNFα-certolizumab complex (heavy chain: cyan; light chain: yellow; heavy chain complementary-determining regions: blue; light chain complementary-determining regions: red); (**C**) Cross-eyed stereoview of the 2fo-fc composite omit map (1.2 σ contour level) at the complementary-determining regions (CDRs) of the free certolizumab Fab fragment, calculated at 1.95 Å resolution (heavy chain: purple; light chain: green); (**D**) Cross-eyed stereoview of the 2Fo-Fc composite omit map (1.2 σ contour level) at the CDRs of the Fab fragment in the TNFα-certolizumab complex, calculated at 2.89 Å resolution (heavy chain: cyan; light chain: yellow).

We determined and refined the crystal structure of human TNFα in complex with the certolizumab Fab fragment at a resolution of 2.89 Å with *R*/*R*_free_ = 0.225/0.265. The crystallographic asymmetric unit contained 3 copies of TNFα-certolizumab Fab complex with a non-crystallographic 3-fold symmetry (Figure 1A). The gel filtration results also indicated a 3:3 molar ratio for TNFα and certolizumab Fab fragment in the complex (data not shown). Almost all residues of TNFα, except those in the EF loop region, were well defined in the electron density map. The dimensions of the trimeric complex of the TNFα-certolizumab Fab fragment were 130 × 130 × 75 Å^3^. When viewed along the 3-fold axis, the trimeric complex had a shape that resembles a three-bladed propeller, with one protomer representing one blade. It consisted of three certolizumab Fab fragments radially bound to a single TNFα homotrimer. The root mean square (rms) deviations between equivalent residues from the protomers of TNFα or the certolizumab Fab molecules in the complex were less than 0.25 Å, as non-crystallographic symmetry restraints were applied during most of the refinement process. The pseudo 2-fold axes of the bound certolizumab Fab fragments relating the heavy and light chains intersected the 3-fold axis of the TNFα homotrimer and had an approximate angle of 40˚ downward from a plane that was perpendicular to the 3-fold axis. When we consider a cell with a TNFα precursor attached, this plane represents the cell membrane (Figure 1A). In this binding orientation, certolizumab not only can bind to soluble TNFα, but also to a TNFα precursor that is not released from the cell membrane. This structural feature is quite consistent with the drug characteristics, which targets both soluble TNFα and transmembrane TNFα [23]. Each TNFα protomer of the trimeric complex adopted a typical β-sandwich with jellyroll topology composed of two five-stranded antiparallel β-sheets [12]. Superimposing TNFα in the TNFα-certolizumab Fab complex with its receptor-bound form (PDB code 3ALQ) yielded an rms deviation of 0.38 Å for all Cα atoms and indicated no significant overall structural changes, except for the conformational change of the DE loop region due to the interaction with certolizumab, which will be described later.

The crystal structure of the uncomplexed certolizumab Fab fragment was also determined and refined to a resolution of 1.95 Å with *R*/*R*_Free_ = 0.147/0.179. The certolizumab Fab presented a canonical immunoglobulin fold and four intramolecular disulfide bonds in the structures of both its uncomplexed form and its TNFα-bound form, as expected. The elbow angle of the certolizumab Fab fragment, defined as the angle subtended by the two pseudo-dyad axes relating the variable and constant domains of the Fab fragment, did not differ between the TNFα-bound and the free certolizumab, despite the intrinsic flexibility of the Fab elbow (Figure 1B). The electron density of the structure of the uncomplexed Fab fragment was clear throughout the entire structure, including in the complementary-determining regions (CDRs) (Figure 1C). Additionally, all CDRs of the structure of the uncomplexed certolizumab Fab fragment had the same conformation as those of this antibody in complex with TNFα, implying that this antibody drug maintains the CDRs in productive binding conformations prior to interactions with TNFα, thereby contributing to the high binding affinity to TNFα (Figure 1B–D).

### 2.2. Interaction between TNF-α and Certolizumab Fab

**Figure 2:**
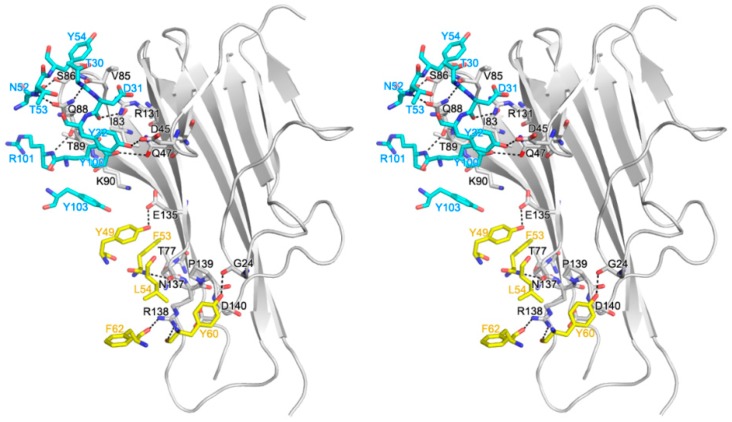
TNFα-certolizumab Fab fragment interface. Cross-eyed stereoview of the detailed TNFα-certolizumab Fab fragment interface. The carbon atoms from TNFα and the heavy and light chains of certolizumab are colored gray, cyan, and yellow, respectively. Hydrogen bonds are indicated with dashed lines.

The interaction of a single Fab fragment of certolizumab with TNFα buried a total solvent-accessible area of 1887 Å^2^, which was larger than a typical protein–protein interface (1560–1700 Å^2^) [38], and thereby contributed to the high affinity between TNFα and certolizumab (Figure 2) [39]. Although TNFα exists as a trimer, the epitope of certolizumab was composed of only residues from a single protomer of TNFα. The certolizumab epitope of TNFα consisted of a number of residues, including _TNFα_G24, _TNFα_D45, _TNFα_Q47, _TNFα_T77, _TNFα_I83, _TNFα_V85, _TNFα_S86, _TNFα_Q88, _TNFα_T89, _TNFα_K90, _TNFα_R131, _TNFα_E135, _TNFα_N137, _TNFα_R138, _TNFα_P139, and _TNFα_D140, and most were located in the DE loop and GH loop regions of TNFα. Several residues on the epitope of certolizumab were also involved in the TNFα–TNFR interaction [35], indicating the mechanism by which certolizumab competitively blocks the TNFα–TNFR interaction.

While all three CDRs from the heavy chain of certolizumab participated in the interaction with TNFα, only one CDR from the light chain, LCDR2, was involved in the TNFα interaction. The interaction of the light chain of certolizumab mediated only by the LCDR2 loop was quite unique; it is generally observed that the LCDR2 region of antibodies is frequently not involved in antigen binding [40]. The paratope of certolizumab consisted of _heavy_T30, _heavy_D31, _heavy_Y32 of HCDR1, _heavy_N52, _heavy_T53, and _heavy_Y54 of HCDR2; _heavy_Y100 _heavy_R101, and _heavy_Y103 of HCDR3; and _light_Y49, _light_F53, _light_L54, _light_Y60, and _light_F62 of LCDR2.

There were 12 hydrogen bonds and no salt bridge interaction between a single protomer of TNFα and the certolizumab Fab fragment, and several residues of TNFα contributed to van der Waals contacts with the certolizumab. The heavy chain of certolizumab interacted with the B′B loop and the G strand as well as the DE loop. The side chain atoms of _TNFα_D45 and _TNFα_Q47 in the B′B loop formed hydrogen bonds with the hydroxyl groups of _heavy_Y32 and _heavy_Y100, respectively. The residues _TNFα_I83, _TNFα_V85, _TNFα_S86, _TNFα_Q88, _TNFα_T89, and _TNFα_K90 of the DE loop were involved in the interaction with certolizumab. The backbone carbonyl groups of _TNFα_S86 and _TNFα_Q88 formed hydrogen bonds with the side chain of _heavy_N52 and the backbone amide group of _heavy_R101, and the side chain of _TNFα_Q88 made two hydrogen bonds with the backbone atoms of _heavy_T30 and _heavy_T53. The side chains of _TNFα_I83, _TNFα_V85, _TNFα_T89, and _TNFα_K90 made van der Waals contacts with the side chains of _heavy_Y100, _heavy_Y54, _heavy_R101, and _heavy_Y103, respectively. The side chain of _TNFα_R131 in the G strand also made a hydrogen bond with the backbone carbonyl group of _heavy_D31. The interaction between the light chain of certolizumab and TNFα was primarily attributed to the GH loop. Several residues in the D strand and AA′′ loop also contributed to the interaction with certolizumab. The residues in the GH loop of TNFα involved in the interaction with certolizumab were _TNFα_E135, _TNFα_N137, _TNFα_R138, _TNFα_P139, and _TNFα_D140. The side chain atom of _TNFα_E135 and the backbone carbonyl group of _TNFα_N137 made hydrogen bonds with the side chain atom of _light_Y49 and the backbone amide group of _light_L54, respectively. _TNFα_R138 made two hydrogen bonds with the backbone carbonyl groups of _light_Y60 and _light_F62. In addition, the backbone carbonyl groups of _TNFα_G24 in the AA´´ loop made a hydrogen bond with the side chain atom of _light_Y60. The residues _light_F53, _light_L54, and _light_Y60 of LCDR2 contributed to van der Waals contacts with the side chains of _TNFα_T77 in the D strand and _TNFα_N137, _TNFα_R138, _TNFα_P139, and _TNFα_D140 in the GH loop.

**Figure 3:**
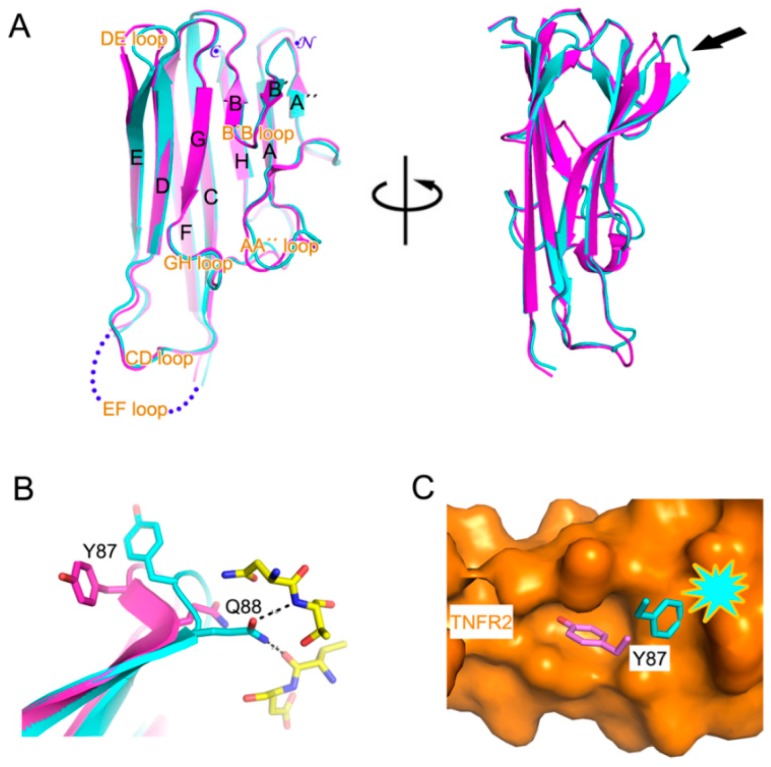
Conformational change of the DE loop. (**A**) Superposition of the TNFα protomers extracted from the TNFα-certolizumab complex (cyan) and the TNFα–TNFR2 complex (purple) in two orientations. The strands, loops, N-, and C-terminus of TNFα are labelled. The arrow indicates the discrepancy of the DE loop conformation; (**B**) Conformational change of the DE loop induced by certolizumab binding. The bidentate hydrogen bond by _TNFα_Q88 with certolizumab (yellow) changes the DE loop conformation; (**C**) Steric collision of _TNFα_Y87 with TNFα is represented in the case of the DE loop conformation altered by certolizumab binding. The surface of TNFR2 is colored orange.

Interestingly, the bidentate hydrogen bond mediated by _TNFα_Q88 induced a conformational change of the DE loop of TNFα (Figure 3A,B). In the structure of TNFα in complex with TNFR2, _TNFα_Y87 of the DE loop was optimally accommodated into a small pocket on the surface of TNFR2 and thereby contributed to the energetics of the TNFα-TNFR2 interaction (Figure 3C) [35]. However, the structural change of the DE loop induced by certolizumab binding was incompatible with TNFR2 binding, as the positional change of _TNFα_Y87 would cause steric collision with TNFR2 (Figure 3B,C). Thus, the neutralizing effect of certolizumab appears to be a consequence of the partial overlap of the epitope with the TNFα-TNFR interface and an antibody-induced conformational change of the DE loop.

### 2.3. Mutagenesis Study of the TNFα-Certolizumab Interface

**Figure 4:**
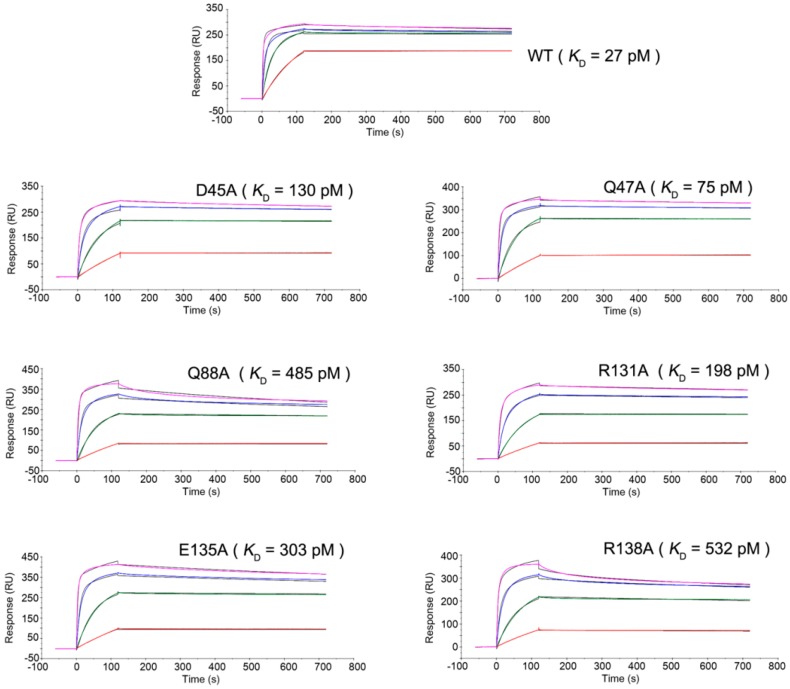
Sensorgrams for the binding kinetics of the TNFα mutants. Surface plasmon resonance analyses of wild-type and mutant TNFα, demonstrating their binding affinities to certolizumab Fab fragments. The concentrations of the wild-type and mutant TNFα for each experiment are 2 (red), 10 (green), 50 (blue), 250 nM (purple).

For the mutagenesis analysis, we selected 6 residues of TNFα whose side chains were involved in the hydrogen bonds with certolizumab; i.e., _TNFα_D45, _TNFα_Q47, _TNFα_Q88, _TNFα_R131, _TNFα_E135, and _TNFα_N137. Each residue was replaced by alanine and the binding affinities of each mutant with certolizumab were measured by surface plasmon resonance to evaluate the effects of these replacements on the interaction with certolizumab (Figure 4). The substitutions of _TNFα_D45, _TNFα_Q47, and _TNFα_R131 with alanine did not substantially affect the binding affinity of TNFα to certolizumab, with decreases in the on-rate constants *k*_on_ of 3–5-fold and similar off-rate constant *k*_off_ values. These results imply that the hydrogen bonds mediated by these residues would only facilitate fast associations between TNFα and certolizumab, but are not important for slow dissociation in order to maintain the stable TNFα-certolizumab complex (Table 1). The replacement of _TNFα_Q88 resulted in a drastic decrease in binding affinity, with an 18-fold higher dissociation constant, *K*_D_, implying that the hydrogen bonds by _TNFα_Q88 play a critical role in the interaction between the DE loop of TNFα and the heavy chain of certolizumab. The substitution of _TNFα_R138 also dramatically increased *K*_D_ by 20-fold, implying the importance of this residue for the interaction between the GH loop of TNFα and the light chain of certolizumab. The critical contribution of the residues _TNFα_Q88 and _TNFα_R138 to the binding affinity can be easily predicted from the structural features of the TNFα-certolizumab interaction, as the side chains of only these two residues made bidentate hydrogen bonds with certolizumab (Figure 2). The hydrogen bond between _TNFα_E135 and _light_Y49 may also play a supplementary role in the energetics of the TNFα-certolizumab interaction as the mutation _TNFα_E135A increased *K*_D_ by 11-fold. Nonetheless, the mutation of a single residue side chain did not result in the complete loss of the TNFα-certolizumab interaction but slightly decreased the binding affinity. This indicates that the interaction surface is very extensive, involving a molecular network rather than individual residues.

## 3. Discussion

TNFα is an important target for the treatment of inflammatory diseases, such as rheumatoid arthritis and inflammatory bowel diseases. Accordingly, five biological agents against TNFα have been approved by the FDA including three monoclonal anti-TNFα full IgG1 antibodies, infliximab, adalimumab, and golimumab; the PEGylated Fab fragment of the anti-TNFα antibody certolizumab pegol; and the extracellular domain of the TNFR2/IgG1-Fc fusion protein etanercept (Table 2). Each shows excellent efficacy, with similar rates of response, although the similarity is somewhat controversial owing to the lack of a head-to-head comparative studies [41].

Comparison of the TNFα interactions of each TNFα blocker can provide a better understanding of the neutralizing mechanism of these anti-TNFα drugs. The structural features of the TNFα-etanercept interface can be deduced from the crystal structure of TNFα in complex with TNFR2, as the TNFα binding part of etanercept is the extracellular domain of TNFR2, implying that the drug exerts neutralizing effects by occupying the receptor binding site of TNFα [35]. The crystal structures of TNFα in complex with infliximab and adalimumab have revealed the epitopes of each antibody drug, showing that they bind to TNFα efficiently and outcompete TNFRs for binding to TNFα, thereby preventing TNFα from functioning in inflammatory diseases [36,37]. However, structural studies of certolizumab pegol and golimumab have not been reported, despite many biochemical and clinical analyses of them. In this study, we report the crystal structure of the soluble trimer of human TNFα in complex with the Fab fragment of the therapeutic antibody certolizumab pegol to understand the antigen-antibody interface and the neutralizing mechanism of this drug. The structure showed that three Fab fragments bind symmetrically to a TNFα trimer. Certolizumab neutralizes TNFα function by partially overlapping with the TNFα-TNFR interface and preventing the conformational rearrangement of the DE loop, which is necessary for TNFR binding.

**Figure 5:**
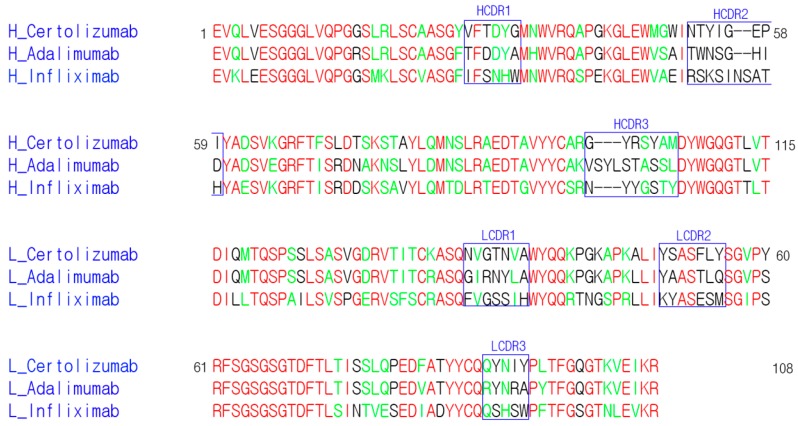
Sequence comparison of the antibodies against TNFα. The CDRs are indicated with boxes and labeled. The residue numbers refer to those in certolizumab. The identical and homologous residues are colored red and green, respectively.

**Figure 6:**
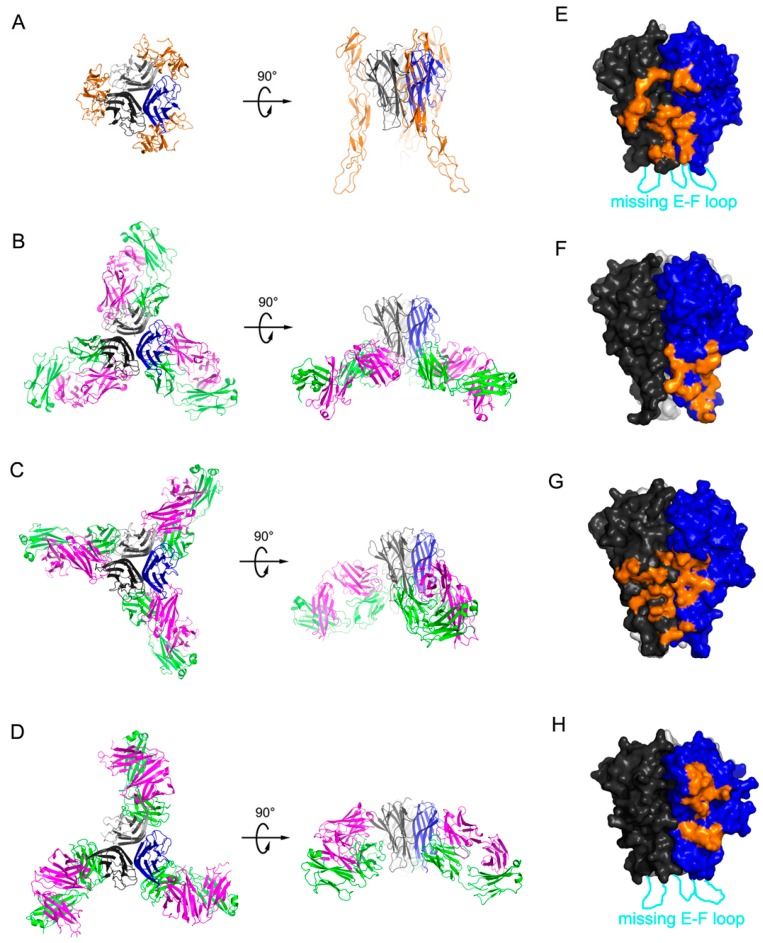
A comparison of the interface between TNFα and the TNFα blockers. (**A**) The structure of the TNFα trimer (black, gray, and blue) in complex with TNFR2 (orange); (**B**) The structure of the TNFα trimer (black, gray, and blue) in complex with the infliximab Fab fragment (heavy chain: purple; light chain: green); (**C**) The structure of the TNFα trimer (black, gray, blue) in complex with the adalimumab Fab fragment (heavy chain: purple; light chain: green); (**D**) The structure of the TNFα trimer (black, gray, blue) in complex with the certolizumab Fab fragment (heavy chain: purple; light chain: green); (**E**) The TNFR2 binding site on the surface of the TNFα trimer (black and blue for each protomer) is colored orange; (**F**) The infliximab epitope on the surface of the TNFα trimer (black and blue for each protomer) is colored orange; (**G**) The adalimumab epitope on the surface of the TNFα trimer (black and blue for each protomer) is colored orange; (**H**) The certolizumab epitope on the surface of the TNFα trimer (black and blue for each protomer) is colored orange. The EF loop, which is missing in the structures of TNFα–TNFR2 and the TNFα-certolizumab complex owing to a lack of interactions, is labeled.

The CDRs of certolizumab have a typical length without an unusual amino acid sequence, according to a Kabat antibody sequence database search [42], which is similar to the other antibodies infliximab and adalimumab (Figure 5). However, comparison of the interactions of certolizumab with other TNFα blockers shows that the epitopes are very different from each other (Figure 6). In the TNFα-adalimumab Fab complex, one Fab fragment of adalimumab interacts with two adjacent TNFα protomers, similar to the TNFα-TNFR2 complex [37]. By contrast, the interactions mediated by the infliximab and certolizumab Fab fragments involve only one protomer of the TNFα homotrimer [36]. The EF loop of TNFα is involved in the interaction with the adalimumab and infliximab Fab fragments. In particular, in the TNFα-infliximab Fab complex, the residues in the EF loop play a crucial role in the antigen-antibody interaction. However, this region in the TNFα-certolizumab Fab complex is completely unobservable in the crystal structure, indicating that the EF loop is flexible and not involved in the interaction with certolizumab, as observed in the structure of the TNFα–TNFR2 complex [35]. It has been reported that the TNFα homotrimer is non-stable at physiological concentrations and slowly dissociates into a monomeric form with reversible trimerization, although the details of this process are not fully elucidated [43,44,45]. Etanercept, adalimumab, and infliximab were found to completely abrogate this monomer exchange reaction in the TNFα homotrimer, whereas certolizumab and golimumab could not prevent it but did slow down the monomer exchange process [39]. In other words, the former three anti-TNFα drugs stabilize the trimeric form of TNFα, whereas the others exhibit no or only slight stabilization. The differences in the monomer exchange behavior of the TNFα blockers are not likely correlated with their binding affinities to TNFα [39]. In the experiment, to measure the affinity of the Fab fragments of the TNFα blockers, the adalimumab Fab fragment, which inhibits the monomer exchange reaction and stabilizes the TNFα homotrimer, had the lowest affinity, whereas the certolizumab Fab fragment had the highest affinity to TNFα [39]. This high affinity of certolizumab Fab may lead to an excellent therapeutic efficacy, similar to those of other bivalent biologics, despite its monovalency originating from the shape of the PEGylated Fab fragment. The differences in TNFα homotrimer stabilization can be explained by the structural features of TNFα in complex with the biologics, described above (Figure 6). Adalimumab and etanercept interact with two neighboring protomers of TNFα simultaneously [35,37], thereby stabilizing interactions between the protomers in the TNFα homotrimer. Although the epitope of infliximab consists of the residues from only one protomer, the antigen-antibody interaction involves the EF loop and leads to its unique conformation [36], which may contribute to the stabilization of the trimeric form of TNFα via the productive communication between the EF loops of the unique conformation in the trimer. On the contrary, the binding of certolizumab is limited to only a single protomer and does not involve the EF loop without influencing its conformation or the interactions between the protomers in the TNFα homotrimer. Based on the monomer exchange behavior of golimumab, which is similar to that of certolizumab, golimumab is expected to bind to an epitope composed of only a single protomer without interacting with the EF loop of TNFα.

## 4. Materials and Methods

### 4.1. Expression and Purification of TNFα

Genes encoding the soluble form of human TNFα (aa 77–233) were subcloned into pET-21a (Addgene, Cambridge, MA, USA). The protein was overexpressed with a C-terminal 6His-tag using plasmid-transformed *E. coli* BL21 (DE3) competent cells. The cells were first grown at 37 °C in Luria-Bertini (LB) medium supplemented with 50 μg·mL^−1^ ampicilin. Protein expression was induced by adding 0.5 mM isopropyl β-d-1-thiogalactopyranoside (IPTG) when the cells reached an optical density at 600 nm of about 0.6, and the cells were grown for 16 h at 18 °C prior to harvesting by centrifugation (3000× *g* for 0.5 h at 4 °C). The cell pellet was resuspended in a lysis buffer (20 mM Tris pH 8.0, 300 mM NaCl, 5 mM β-mercaptoethanol) and disrupted by sonication on ice. After the crude lysate was centrifuged (25,000× *g* for 1 h at 4 °C), the supernatant containing soluble was applied to the HisTrap HP column (GE Healthcare Life Sciences, Marlborough, MA, USA) and washed with five column volumes of wash buffer (20 mM Tris pH 8.0, 300 mM NaCl, 5 mM β-mercaptoethanol, 50 mM imidazole). The protein was then eluted with elution buffer (20 mM Tris pH 8.0, 300 mM NaCl, 5 mM β-mercaptoethanol, 400 mM imidazole). The eluted protein was concentrated for gel filtration chromatography using a HiLoad 16/60 Superdex 200 pg column (GE Healthcare Life Sciences). The column had previously been equilibrated with gel filtration buffer (20 mM Tris pH 8.0, 300 mM NaCl). The protein purity was evaluated by SDS–PAGE.

### 4.2. Expression and Purification of the Certolizumab Fab

The DNA sequence for the Fab fragment of certolizumab was synthesized after codon-optimization for expression in *E. coli* (Bioneer, Inc., Daejon, Korea). The sequences for the heavy chain and the light chain were cloned into a modified pBAD vector, containing the STII signal sequence in each chain for periplasmic secretion and a C-terminal 6His-tag in the heavy chain [46]. The plasmid pBAD-certolizumab Fab fragment was transformed into *E. coli* Top10F (Invitrogen, Carlsbad, CA, USA). The cells were grown at 37  °C in LB medium supplemented with 50 μg·mL^−1^ ampicillin. At an OD600 of 1.0, the protein expression was induced with 0.2% arabinose and cells were grown at 30  °C for 15  h. The cells were harvested by centrifugation, re-suspended in a lysis buffer (20  mM Tris, pH 8.0, 200  mM NaCl), and lysed by sonication on ice. After removing cell debris by centrifugation (25,000× *g* for 0.5  h at 4  °C), the supernatant containing soluble protein was applied to the HisTrap HP column (GE Healthcare Life Sciences) and washed with five column volumes of wash buffer (20  mM Tris, pH 8.0, 300  mM NaCl, 50  mM imidazole). The protein was then eluted with elution buffer (20  mM Tris pH 8.0, 300  mM NaCl, 400  mM imidazole). The eluted protein was concentrated for gel filtration chromatography using a HiLoad 16/60 Superdex 200  pg column (GE Healthcare Life Sciences). The column had previously been equilibrated with gel filtration buffer (20 mM Tris pH 8.0, 300  mM NaCl). The elution profile of the protein showed a single major peak and the protein quality was evaluated by reducing and nonreducing SDS–PAGE.

### 4.3. Crystallization and Structure Determination of the Certolizumab Fab

Gel-filtration fractions containing the certolizumab Fab fragment were concentrated to 10 mg·mL^−1^ in 20 mM Tris, pH 8.0, and 300 mM NaCl. Crystals were grown using a hanging-drop vapor diffusion with a reservoir solution containing 0.1 M Bis-Tris, pH 5.5, 0.2 M ammonium sulfate, and 25% PEG3350 at 20 °C within a week. Crystals were cryoprotected by brief immersion in a well solution, supplemented with 20% glycerol, and flash frozen in liquid nitrogen. X-ray diffraction data were collected at 100 K on beamline 5C of the Pohang Light Source (PLS) (Pohang, Korea). The crystals belonged to space group *P*2_1_2_1_2_1_ (*a* = 58.33, *b* = 63.70, *c* = 161.41 Å) with one copy in the asymmetric unit. X-ray diffraction data were collected to a resolution of 1.95 Å, integrated, and scaled using HKL2000 (HKL Research, Charlottesville, VA, USA). The structure was solved by molecular replacement using a Phaser [47] with a structure of the Fab fragments that has high sequence identities with certolizumab Fab fragments (PDB code 4DKF, chains H and L). Due to the intrinsic elbow flexibility of a Fab fragment, the Fv region and the other region including the CH1 and CL domains were separated when used as a search model. At this point, the electron density corresponding certolizumab was prominent. Iterative rounds of refinement were done using PHENIX [48] with manual inspection using COOT [49]. Statistics for data collection and refinement can be found in Table 3. All structure figures were prepared using PyMOL [50].

### 4.4. Crystallization and Structure Determination of the TNFα-Certolizumab Fab Complex

Purified TNFα and certolizumab Fab were mixed in a 1:1 molar ratio and incubated for 1 h at 4 °C before being subjected to size exclusion chromatography using a HiLoad 16/60 Superdex 200 pg column equilibrated with 20 mM Tris, pH 8.0, and 300 mM NaCl. Gel-filtration fractions containing the TNFα-certolizumab Fab complex were concentrated to 7 mg·mL^−1^ in 20 mM Tris, pH 8.0, and 300 mM NaCl. Crystals were grown using hanging-drop vapor diffusion with a reservoir solution containing 0.1 M 3-(cyclohexylamino)-1-propanesulfonic acid pH 5.6, 0.2 M lithium sulfate, and 1.5 M ammonium sulfate at 20 °C within 20 days. Crystals were cryoprotected by brief immersion in the well solution, supplemented with 25% ethylene glycol, and flash frozen in liquid nitrogen. X-ray diffraction data were collected at 100 K on beamline 7A of the Pohang Light Source (PLS) (Pohang, Korea). The crystals belonged to space group *C*2 (*a* = 148.59, *b* = 207.22, *c* = 112.63 Å, β = 118.81˚) with three copies in the asymmetric unit. X-ray diffraction data were collected to a resolution of 2.89 Å, integrated, and scaled using HKL2000 (HKL Research, Charlottesville, VA, USA). The structure was solved by molecular replacement using Phaser with a structure of the free certolizumab Fab fragment and human TNFα (PDB code 1TNF). Due to the intrinsic elbow flexibility of a Fab fragment, the Fv region and the other regions, including the CH1 and CL domains, were separated when the structure of the free certolizumab Fab fragment was used as a search model. At this point, the electron density corresponding to the TNFα-certolizumab Fab Complex was prominent. Iterative rounds of refinement were done using PHENIX with manual inspection using COOT. Statistics for data collection and refinement can be found in Table 1.

### 4.5. Binding Kinetics of the TNFα WT and Mutants

Site-directed mutants of TNFα, including _TNFα_D45A, _TNFα_Q47A, _TNFα_Q88A, _TNFα_R131A, _TNFα_E135A, and _TNFα_R138A, were created with the QuickChange Kit (Agilent Technologies, Santa Clara, CA, USA) and confirmed by DNA sequencing. The mutant proteins were expressed and purified as described for wild-type TNFα. Approximately 1000 response units of the certolizumab Fab fragment were immobilized on the surface of a CM-5 chip (GE Healthcare Life Sciences) via amine coupling reactions, as described in the manufacturer’s instructions. Purified wild-type and the mutants of TNFα were serially diluted to concentrations ranging from 2 nM to 250 nM using PBS buffer and flowed through the chip. A BIAcore T100 instrument (GE Healthcare Life Sciences, Marlborough, MA, USA) was operated at 25 °C using PBS buffer as a running buffer. The bound TNFα was removed with 10 mM glycine (pH 2.0) at the end of each cycle while retaining the surface integrity for chip regeneration. Sensorgrams were locally fitted and the dissociation constants (*K*_d_) were calculated with the analysis software, BIAevaluation (GE Healthcare Life Sciences, Marlborough, MA, USA).

### 4.6. Accession Number

The coordinates and structure factors for the crystal structures of the free certolizumab Fab fragments and the complex of TNFα-certolizumab Fab fragments have been deposited in the Protein Data Bank under accession codes 5WUV and 5WUX, respectively.

## 5. Conclusions

In summary, the elucidation of the crystal structure of TNFα in complex with the Fab fragments of certolizumab pegol sheds light on the molecular mechanism underlying the therapeutic activity of this antibody drug. In addition, the precise epitope revealed by the present complex structure could provide useful information for the improvement of the current biological agents against TNFα for the treatment of inflammatory autoimmune diseases.

## Figures and Tables

**Table 1:** Binding kinetics of the TNFα mutants with certolizumab Fab fragments. WT: Wild-type.

TNFα	*K*_on_ (M^−1^·s^−1^)	*K*_off_ (s^−1^)	*K*_D_ (M)
WT	1.97 × 10^6^	5.40 × 10^−5^	2.74 × 10^−11^
D45A	5.86 × 10^5^	7.64 × 10^−5^	1.30 × 10^−10^
Q47A	5.83 × 10^5^	4.39 × 10^−5^	7.53 × 10^−11^
Q88A	4.76 × 10^5^	2.31 × 10^−4^	4.85 × 10^−10^
R131A	3.74 × 10^5^	7.42 × 10^−5^	1.98 × 10^−10^
E135A	4.60 × 10^5^	1.40 × 10^−4^	3.04 × 10^−10^
R138A	4.52 × 10^5^	2.41 × 10^−4^	5.32 × 10^−10^

**Table 2:** Approved biologics against TNF-α.

Drug	Trade Name	Type	Approval Date	
			FDA	EMA
Etanercept	Enbrel	TNFR2 extracellular portion Fc fusion	1998	2000
Infliximab	Remicade	Chimeric murine/human IgG1	1998	1999
Adalimumab	Humira	Fully Human IgG1	2005	2003
Certolizumab-pegol	Cimzia	Humanized, PEGylated Fab’	2008	2009
Golimumab	Simponi	Fully Human IgG1	2009	2009

FDA, Food and Drug Administration; EMA, European Medicine Agency.

**Table 3:** Data collection and refinement statistics.

	Certolizumab Fab	TNFα-Certolizumab Fab
Data Collection		
X-ray source	PLS 5C	PLS 7A
Wavelength (Å)	1.0000	1.0000
Space group	*P*2_1_2_1_2_1_	*C*2
Cell dimensions		
*a*, *b*, *c* (Å)	58.33, 63.70, 161.41	148.59, 207.22, 112.63
α, β, γ (°)	90, 90, 90	90, 118.81, 90
Resolution (Å)	1.95 (1.98–1.95) *	2.89 (2.95–2.89)
*R*_sym_ (%)	7.8 (29.7)	8.1 (48.6)
*I/σI*	58.1 (3.1)	19.6.1 (2.3)
Completeness (%)	98.2 (85.6)	95.2 (94.5)
Redundancy	5.9 (2.5)	2.9 (2.6)
Refinement		
Resolution (Å)	1.95	2.89
No. reflections	43749	63355
*R*_work_/*R*_free_ (%)	14.7/17.9	22.5/26.5
No. atoms		
Protein	3290	12793
Water	607	0
R.m.s. deviation		
Bond lengths (Å)	0.007	0.006
Bond angles (˚)	1.060	1.278
Ramachandran		
Favored (%)	98.37	95.04
Allowed (%)	1.63	4.47
Outlier (%)	0.00	0.49
PDB code	5WUV	5WUX

* Values in parentheses are for the outer resolution shell.
